# DFDBA with or without allogenic human dental pulp derived mesenchymal stem cells in treatment of mandibular class II furcation defects

**DOI:** 10.6026/973206300223357

**Published:** 2026-06-30

**Authors:** Kousain Sehar, Abhima Kumar, Sabiha Parwaiz, Shrey Shaileshbhai Patel, Mohd Irfan, Kamalnathgandhi M

**Affiliations:** 1 Department of Periodontology and Oral Implantology, Indira Gandhi Government Dental College & Hospital, Jammu, Jammu and Kashmir, India, 180005

**Keywords:** Dental pulp stem cells (DPSCs), demineralized freeze-dried bone allograft (DFDBA), regeneration, tissue engineering

## Abstract

It is a challenging task to regenerate the periodontium at the furcation defect. Tissue engineering techniques using stem cells have gained importance in medical field in recent years. Various methods are tried to treat furcation defects. Therefore, it is of interest to assess DFDBA with or without allogenic human dental pulp derived mesenchymal stem cells in treatment of mandibular class II furcation defects. Hence, 28 systemically healthy individuals with at least one mandibular class II furcation defect were divided into 2 groups. Group A (n=14) patients were treated with DFDBA alone and Group B (n= 14) were treated with combination of DFBDA and allogenic dental pulp derived mesenchymal stem cells. Clinical parameters i.e., vertical relative attachment level (V-RAL), vertical probing depth (VPD), horizontal probing depth (HPD) and gingival recession (GR) were recorded at baseline and 6-months post-operatively. DPSCs with DFDBA did not show any statistical significance in both clinical and hard tissue outcome parameters.

## Background:

The therapy of furcation involvement depends primarily on the extent of disease and the configuration of the defect. Furcation defects have been classified by different authors based on horizontal measurement of attachment loss, horizontal and vertical measurement of attachment loss or a combination of these findings [1]. Treatment of furcation defects begin with the non-surgical periodontal therapy. However, scaling and root planning (SRP) alone is not sufficient as a treatment strategy for furcation defects because of anatomic limitations [2]. In teeth with furcation involvement especially class II regeneration of lost periodontium is desirable for long term stability of teeth [3]. The ultimate goal of periodontal regeneration aims at the restoration of periodontium to its original form and function. Due to the complex structure of periodontium, consisting of both hard and soft tissues, its entire regeneration has always been a challenge [4]. Various regenerative approaches have been advocated till date to achieve regeneration such as the bone grafts (autologous, allogenic, xenografts, alloplasts), membranes and platelet concentrates [5]. Among the various regenerative materials attempted, bone grafts have been widely evaluated for regeneration. Although autologous grafts are ideal, because of the limited amount of intraoral graft procurement and the need for a second surgical site for bone graft harvesting, allogenic bone graft materials have emerged as viable alternatives [6]. Demineralized freeze dried bone allograft (DFDBA) is one such allogenic bone graft material and is widely used compared to other allogenic grafts (fresh frozen bone, freeze dried bone allograft). DFDBA has the added advantage of releasing bone inductive proteins called bone morphogenic proteins (BMP's) that makes the graft both osteoconductive as well as osteoinductive [7]. Since last few decades, research has been focusing on the possible application of stem cells for restoring and regeneration of dental and dentofacial structures. Recently, the use of stem cells for regeneration through tissue engineering has been gaining increasing attention [8]. Tissue engineering involves a triad of signaling molecules, scaffolds and stem cells which are essential for true regeneration to take place when appropriate environmental conditions are provided [9]. Therefore, it is of interest to compare the efficacy of demineralized freeze dried bone allograft with or without allogenic human dental pulp derived mesenchymal stem cells in the treatment of mandibular class II furcation defects.

## Materials and Methodology:

The present study was designed as a randomized, controlled, clinical two arm parallel study to evaluate the efficacy of DFDBA with or without allogenic human pulp derived mesenchymal stem cells (DPSCs) in the treatment of human mandibular class II furcation defects. Inclusion criteria were; systemically healthy patients presenting with at least one mandibular molar tooth with class II furcation defect. Exclusion criteria were; patients who underwent periodontal therapy in past 3 months, teeth with concomitant angular bone defects and teeth with mobility ≥ grade II. Patients with smoking habit, chronic alcoholics and who on medication were excluded from the study. All the recruited patients were explained about the study protocol and written informed consent was taken prior to commencement of the study. Total 28 patients were randomly assigned into two equal groups. Group A: 14 patients who received OFD with placement of DFDBA in furcation defect. Group B: 14 patients who received OFD with placement of DFDBA along with allogenic human DPSCs in furcation defect. A perforated stock metal impression tray was selected for each patient. An irreversible hydrocolloid impression material (alginate) was used for making mandibular arch impressions. Study casts were prepared for each patient followed by fabrication of a customized acrylic occlusal stent on the study model. The apical border of the stent was used as a fixed reference point (AS) to take clinical measurements. The stents were made such that there will be prominent mid buccal or mid lingual extensions towards the furcation defect aspect. One vertical groove was prepared in the stent for reproducible alignment of a periodontal probe. Vertical measurements were recorded using the UNC-15 probe (University of North Carolina probe) and horizontal measurements using Nabers Q2N probe. As the Nabers Q2N probe has colour coded regions from 3-6mm and 9-12mm, the defect depth was recorded such that when >half of 3-6mm band is submerged it is recorded as 4mm, >half but <two-third of colour coded band is submerged it is recorded as 5mm and if >two-third or near the end of coded area it is recorded as 6mm.The use of the customized occlusal stent is more reliable as a fixed reference point and for reproducibility of angulations for clinical calibrations at each site over the entire duration of the study. The vertical clinical attachment level, horizontal and vertical probing depth and gingival recession were calculated from a fixed reference point on the stent. All the customized acrylic stents were stored on the study models throughout the study period to minimize distortion. For soft tissue assessment, a UNC-15 probe and Nabers probe were used long with indices. Modified gingival index (MGI), (Lobene *et al.* 1986) [10], Full mouth plaque scores (FMPS), (O'Leary *et al.* 1972) [11] and Full mouth bleeding scores (FMBS), (Cortellini *et al.* 1993) [12] indices were used for analysis. Hard tissue assessments were done at baseline and after 6-months post-surgery. Hard tissue measurements were taken through bone sounding only after adequate local anaesthesia is administered. All patients advised for proper oral hygiene measure. The obtained data was statistically evaluated using SPSS version 25.0IBM with Wilcoxon signed rank test, Mann-Whitney U test, t test and ANOVA test.

## Results:

Baseline clinical defect characteristics between group A and group B were statistically not significant indicating that both groups were evenly matched at the beginning of the study. Patients of both groups had an uneventful healing and there were no local or systemic complications or any adverse effects reported post-surgery (Table 1). Intra-group comparison of mean V-RAL, VPD, HPD and GR from baseline to 6 months for group A (p<0.05) and group B (p<0.05) were statistically significant except for GR (Table 2, Table 3). Intra group comparison of VDF-P and HDF-P from baseline to 6-months post- operatively for both group A (p<0.05) and group B (p<0.05) were statistically significant. Inter group comparison of VFB and HFB from baseline to 6 months post-operatively between both the groups revealed no statistically significant difference (pwas >0.05) (Table 4). Inter group analysis of hard tissue outcome parameters (VFB and HFB) between groups was non-significant (Table 5). The probing bone level measurements versus open bone level measurements at base line were significant (Table 6). Furcation sites according to Hamp *et al.* [12] at base line and 6-months post-operatively for both group is shown in Table 7. It indicates the improvement. Table 8 indicates Intra-group analysis of MGI. The values were not statistically significant at baseline to 6 months of follow up. Intra-group analysis of FMPS was non-significant (Table 9, Table 10, Table 11).

## Discussion:

Periodontal therapy aims not only towards the arrest of periodontal disease progression, but also for the regeneration of structures hat are lost to disease activity. Conventional periodontal therapy such as open flap debridement provides a reliable method for accessing root surfaces, reducing the depth of periodontal pockets and to attain improved periodontal form and architecture [13]. However, these techniques offer only limited potential towards regeneration of the tissues that were destroyed due to previous disease activity. With the advances in the field of periodontics, the surgical procedures now-a-days are incorporating novel techniques such as tissue engineering aiming at a greater predictable regeneration of periodontal tissues and functional attachment [14]. Although there is adequate literature on the use of DFDBA in regeneration of class II furcation defects, there is a paucity of evidence regarding the use of allergenic dental pulp derived mesenchymal stem cells in the periodontal regenerative procedures especially furcation defects [15]. It is presumed or hypothesized that DFDBA acts as a scaffold material and the undifferentiated mesenchymal stem cells when supplemented with DFDBA will be acted upon by the local signaling molecules which may result in complete and true regeneration [16, 17]. Biradar *et al.* assessed the clinical and radiographic outcomes of using demineralized freeze-dried bone allograft (DFDBA) alone versus its combination with doxycycline hyclate loaded proniosomal gel in the management of class II furcation defects. They concluded that, the adjunctive use of doxycycline hyclate-loaded proniosomal gel with DFDBA improved periodontal regeneration [18]. Basireddy *et al.* evaluated the ability of platelet-rich fibrin (PRF) to augment the regenerative effects exerted by demineralized freeze-dried bone allograft (DFDBA) in the treatment of mandibular degree II furcation defects. They concluded that, PRF seems to favor soft-tissue healing but has no additional benefit in bone regeneration when used in combination with DFDBA [19]. We found that, DPSCs with DFDBA did not show any statistical significance in both clinical and hard tissue outcome parameters.

## Conclusion:

Adjunctive use of allogenic human DPSCs with DFDBA did not show any statistical significance in both clinical and hard tissue outcome parameters. *In vivo* regeneration of periodontal defects is highly technique sensitive which needs to be addressed in future. At this point of time, there is insufficient literature evidence in human studies using allogenic DPSCs with positive results that are statistically significant in periodontal regeneration. Further studies with larger sample size and longer follow-up period are needed to provide additional insight into this novel combination.

## Advancement to knowledge:

Regenerative dentistry has advanced significantly with the use of allogenic Human Dental Pulp-derived Mesenchymal Stem Cells (hDPSCs), particularly for the treatment of difficult periodontal diseases such as mandibular class II furcation defects. This strategy provides a viable, less invasive substitute for conventional grafting techniques by utilizing the high proliferative capacity and immunomodulatory potential of DPSCs produced from healthy, removed teeth (such as wisdom teeth).

## Figures and Tables

**Table 1:** Analysis of demographic data, clinical characteristics of furcation defects and indices among group A and group B

**Baseline parameters**	**Group A (n=14)**	**Group B (n=14)**	**p-value**
Age(in years)	36.28±7.89	40.07±9.49	0.402(NS)
Gender(Mv/sF)	9v/s5	7v/s7	-
V-RAL(in mm)	8.57±0.94	8.93±0.99	0.469(NS)
VPD(in mm)	5.29±0.73	5.14±0.86	0.608(NS)
HPD(in mm)	5.36±0.49	5.50±0.52	0.453(NS)
GR(in mm)	0.36±0.49	0.43±0.65	0.891(NS)
VDF-P(in mm)	6.29±0.73	6.14±0.86	0.608(NS)
HDF-P(in mm)	6.36±0.49	6.50±0.52	0.453(NS)
Defect location			
Buccaldefect	5	6	
Lingual defect	9	8	
MGI	0.47±0.10	0.47±0.14	0.946(NS)
FMPS	18.30±1.77	18.04±1.76	0.701(NS)
FMBS	17.80±2.08	17.18±2.21	0.454(NS)
S: Statistically
significant (p≤0.05),
NS: No statistical
significance (pwas >0.05)

**Table 2:** Intra group analysis of primary clinical parameters for group A and B using Wilcoxon signed rank test

**Group**	**Parameter**	**Baseline Mean ±SD(in mm)**	**6-monthspost- operative Mean ±SD(in mm)**	**p-value**
	V-RAL	8.57 ±0.94	5.64 ±0.93	0.001(S)
	VPD	5.29 ±0.73	2.57 ±0.65	0.001(S)
Group A	HPD	5.36 ±0.49	2.36 ±0.49	<0.001(S)
	GR	0.36 ±0.49	0.43 ±0.51	0.317(NS)
	V-RAL	8.93 ±0.99	5.79 ±1.25	0.001(S)
	VPD	5.14 ±0.86	2.50 ±0.52	0.001(S)
Group B	HPD	5.50 ±0.52	2.43 ±0.51	<0.001(S)
	GR	0.43 ±0.65	0.36 ±0.49	0.317(NS)
S: Statistically
significant (p≤0.05),
NS: No statistical
significance (pwas >0.05)

**Table 3:** Inter-group analysis of clinical outcome parameters between group A and group B using the Mann-Whitney U test

**Parameters**	**Group A Mean ±SD(in mm)**	**Group Mean ±SD(in mm)**	**p-value**
V-CAG	2.93 ±0.92	3.21 ±0.69	0.259(NS)
VPD-R	2.71 ±0.47	2.64 ±0.74	0.569(NS)
HPD-R	3.00 ±0.00	3.00 ±0.55	1.00 (NS)
GR	0.07 ±0.27	0.07 ±0.26	1.00 (NS)
Statistically
significant (p≤0.05),
NS: No statistical
significance (pwas >0.05)

**Table 4:** Intra group analysis of hard tissue parameters for group A and B using Wilcoxon signed rank test

**Group**	**Parameter**	**Baseline Mean ±SD (in mm)**	**6monthspost- operatively Mean ±SD (in mm)**	**p-value**
	VDF-P	6.28 ±0.73	3.57 ±0.65	0.001(S)
Group A	HDF-P	6.36 ±0.49	3.36 ±0.49	<0.001(S)
Group B	VDF-P	6.14 ±0.86	3.50 ±0.52	0.001(S)
	HDF-P	6.50 ±0.52	3.36 ±0.49	0.001(S)
Statistically
significant (p≤0.05),
Sino statistical
significance (pwas >0.05)

**Table 5:** Inter group analysis of hard tissue outcome parameters between group A and group B using Mann-Whitney U test

**Parameters**	**Group A Mean ±SD (in mm)**	**Group B Mean ±SD (in mm)**	**p-value**
VFB	2.71 ±0.47	2.79 ±0.69	0.873(NS)
HFB	3.00 ±0.00	3.21 ±0.58	0.147(NS)
Statistically significant (p≤0.05),
NS: statistically not significance (pwas >0.05)

**Table 6:** Comparison of probing bone level measurements versus open bone level measurements at base line

**Parameters**	**Probing bone level measurements in mm)**	**Open bone level measurements in mm)**	**Difference (in mm)**	**Correlation coefficient (rho)**
Vertical				0.671
Depth of furcation	6.21 ±0.79	6.50 ±0.64	0.29	(p-.001)
defect (n=28)				(S)
Horizontal				0.596
Depth of furcation	6.43 ±0.50	6.68 ±0.48	0.25	(p-.001)
defect (n=28)				(S)
Statistically significant (p≤0.05),
NS: statistically not significance (pwas >0.05)

**Table 7:** Number of group A and group B furcation sites according to Hamp *et al.* [12] at base line and 6-months postoperatively.

**Classification**	**Group A**		**Group B**
	Baseline	6 months	Baseline	6 months
Complete furcation closure	0	0	0	0
Class I(<3mm)	0	9	0	9
Class II(was >3mm)	14	5	14	5
Class III(through and through)	0	0	0	0

**Table 8:** Intra-group analysis of MGI for group A and group B using one-way repeated measures of ANOVA test

**Group**	**MGI**	**%Mean ±SD**	**p-value**	**Post-hoc analysis**
Group A (n=14)	MGI baseline	0.47 ±0.10	0.474 (NS)	NS
	MGI3 months	0.45 ±0.13		
	MGI6 months	0.44 ±0.12		
Group B (n=14)	MGI Baseline	0.47 ±0.14	0.485 (NS)	NS
	MGI3 months	0.43 ±0.16		
	MGI6 months	0.41 ±0.16		
Statistically
significant (p≤0.05),
Sino statistical
significance (pwas >0.05)

**Table 9:** Intra-group analysis of FMPS for group A and group B using one- way repeated measures of ANOVA test

**Group**	**FMPS**	**%Mean ±SD**	**p-value**	**Post-hoc analysis**
	FMPS baseline	18.30 ±1.77		
Group A (n=14)	FMPS3 months	18.36 ±2.85	0.690 (NS)	
	FMPS6 months	17.64 ±2.56		NS
	FMPS Baseline	18.04 ±1.76		
Group B (n=14)	FMPS3 months	18.87 ±3.14	0.515 (NS)	
	FMPS6 months	17.69 ±2.77		NS
Statistically
significant (p≤0.05),
Sino statistical
significance (pwas >0.05)

**Table 10:** Intra-group analysis of FMBS for group A and group B using one- way repeated measures of ANOVA test

**Group**	**FMBS**	**%Mean ±SD**	**p-value**	**Post-hoc analysis**
Group A (n=14)	FMBS baseline	17.80 ±2.08	0.012 (NS)	Bwas >6-months (S)
	FMBS3 months	16.46 ±2.49		
	FMBS6 months	14.99 ±2.94		
Group B (n=14)	FMBS Baseline	17.18 ±2.21	0.068 (NS)	NS
	FMBS3 months	16.14 ±2.73		
	FMBS6 months	14.75 ±3.39		
S: Statistically
significant (p≤0.05),
NS: No statistical
significance (pwas >0.05)

**Table 11:** Inter-group analysis of FMPS, FMBS and MGI group A and group B using independent sample t test.

**Indices**	**Group A %Mean ±SD**	**Group B %Mean ±SD**	**p-value**
MGI Baseline	0.47 ±0.10	0.47 ±0.14	0.946(NS)
MGI3 months	0.45 ±0.13	0.43 ±0.16	0.839(NS)
MGI6 months	0.44 ±0.12	0.41 ±0.16	0.571(NS)
FMPS Baseline	18.30 ±1.77	18.04 ±1.76	0.701(NS)
FMPS3 months	18.36 ±2.85	18.87 ±3.14	0.804(NS)
FMPS6 months	17.64 ±2.56	17.69 ±2.77	0.946(NS)
FMBS Baseline	17.80 ±2.08	17.18 ±2.21	0.454(NS)
FMBS3 months	16.46 ±2.49	16.14 ±2.73	0.769(NS)
FMBS6 months	14.99 ±2.94	14.75 ±3.39	0.874(NS)
Statistically
significant (p≤0.05),
NS: No statistical
significance (pwas >0.05)
